# Clinicopathological Characteristics and Mutations Driving Development of Early Lung Adenocarcinoma: Tumor Initiation and Progression

**DOI:** 10.3390/ijms19041259

**Published:** 2018-04-23

**Authors:** Kentaro Inamura

**Affiliations:** Division of Pathology, The Cancer Institute, Japanese Foundation for Cancer Research, 3-8-31 Ariake, Koto-ku, Tokyo 135-8550, Japan; kentaro.inamura@jfcr.or.jp; Tel.: +81-3-3570-0111 (ext. 5604); Fax: +81-3-3570-0558

**Keywords:** CDKN2A (p16), de novo pathway, HNF4A, immunohistochemistry, molecular pathological epidemiology, NKX2-1 (TTF-1) amplification, Napsin A, Noguchi classification, terminal respiratory unit (TRU)-lineage, TP53 (p53)

## Abstract

Lung cancer is the leading cause of cancer-related deaths worldwide, with lung adenocarcinoma representing the most common lung cancer subtype. Among all lung adenocarcinomas, the most prevalent subset develops via tumorigenesis and progression from atypical adenomatous hyperplasia (AAH) to adenocarcinoma in situ (AIS), to minimally invasive adenocarcinoma (MIA), to overt invasive adenocarcinoma with a lepidic pattern. This stepwise development is supported by the clinicopathological and molecular characteristics of these tumors. In the 2015 World Health Organization classification, AAH and AIS are both defined as preinvasive lesions, whereas MIA is identified as an early invasive adenocarcinoma that is not expected to recur if removed completely. Recent studies have examined the molecular features of lung adenocarcinoma tumorigenesis and progression. *EGFR*-mutated adenocarcinoma frequently develops via the multistep progression. Oncogene-induced senescence appears to decrease the frequency of the multistep progression in *KRAS*- or *BRAF*-mutated adenocarcinoma, whose tumor evolution may be associated with epigenetic alterations and kinase-inactive mutations. This review summarizes the current knowledge of tumorigenesis and tumor progression in early lung adenocarcinoma, with special focus on its clinicopathological characteristics and their associations with driver mutations (*EGFR*, *KRAS*, and *BRAF*) as well as on its molecular pathogenesis and progression.

## 1. Introduction

Lung cancer is the leading cause of cancer-related deaths worldwide, with adenocarcinoma representing the most prevalent subtype. As most cases of lung adenocarcinoma are found at advanced stages, even with regular radiographic screening, improved detection of early lung adenocarcinoma at a curable stage would decrease lung cancer-related deaths and avoid costly treatments. Over the past several decades, novel strategies based on newly identified genomic features of these cancers have provided us with refined risk assessments, early detection methods, and therapeutic interventions for lung cancer; however, the impact of these strategies is limited by inadequate knowledge of the biological features of lung cancer, especially the processes of tumorigenesis and tumor progression at early stages. Recent studies have increased our understanding of the genomic features of lung adenocarcinoma in terms of its initiation and progression. The most prevalent subset of adenocarcinoma is believed to develop stepwise from atypical adenomatous hyperplasia (AAH) to adenocarcinoma in situ (AIS), to minimally invasive adenocarcinoma (MIA), and finally, to overt invasive adenocarcinoma with a lepidic pattern.

The recently updated World Health Organization (WHO) classification [1,2], which is based on the accumulated molecular features of cancer, has adopted this stepwise continuum of lung adenocarcinoma tumorigenesis and progression. Considering that this process begins with AAH (the precursor of adenocarcinoma), the 2015 WHO classification divides adenocarcinoma into AIS (preinvasive lesion), MIA, and overt invasive adenocarcinoma, according to the extent of invasiveness [1].

AAH, AIS, and MIA are precursors of overt invasive adenocarcinoma with a lepidic pattern; therefore, a greater understanding of their development may elucidate the underlying mechanisms of tumor evolution in early lung adenocarcinoma. An analysis of these mechanisms will allow the development of more effective prevention and therapeutic strategies. This review presents the current knowledge of the processes of tumorigenesis and progression in early lung adenocarcinoma, with a focus on its clinicopathological characteristics and their associations with driver mutations (*EGFR*, *KRAS*, and *BRAF*).

## 2. Clinicopathological Characteristics

The stepwise continuum of AAH to AIS, to MIA, to overt invasive adenocarcinoma with a lepidic pattern, has been recognized as a potential model of lung adenocarcinoma development. This continuum is supported not only by the morphological appearance of the tumor but also by the sequence of the molecular processes involved. Both cellular and structural atypia increase along this stepwise continuum. Noguchi et al. classified small (less than or equal to 2 cm) peripheral adenocarcinomas into six types (the Noguchi classification; type A–F) based on histological tumor growth patterns. In the Noguchi classification, early adenocarcinomas with lepidic patterns are classified as type A, B, or C [3], representing the stepwise progression of lung adenocarcinoma [3,4]. Type A adenocarcinoma, corresponding to AIS, exhibits lepidic growth with a relatively thin stroma. Type B adenocarcinoma, corresponding to the sclerosing variant of AIS, exhibits lepidic growth with septal widening due to alveolar collapse. Type C adenocarcinoma, corresponding to MIA, represents localized adenocarcinoma with lepidic growth and foci of active fibroblast proliferation [3,4]. This section provides an overview of the clinicopathological features of AAH, AIS, and MIA.

### 2.1. AAH (Atypical Adenomatous Hyperplasia)

AAH has been uniquely recognized as a potential lesion from which lung adenocarcinoma arises and is usually undetectable by imaging techniques. AAH is typically identified incidentally during the examination of surgical specimens harboring a malignant tumor [1]. AAH is a local, slow-growing lesion. On computed tomography (CT), AAH, when detectable, manifests as a small, ground-glass nodule (GGN) with no solid component [5,6,7]. The incidence of AAH is high in surgically resected lung tissue that harbors lung cancer, especially lung adenocarcinoma [8,9]. Among adenocarcinomas, AAH frequently coexists with AIS [9]. This higher risk of AAH in lung tissue harboring adenocarcinoma may be explained by field cancerization [10,11,12], which suggests that normal lung tissue is at risk of developing adenocarcinoma because it possesses genetic features that make the tissue vulnerable to the development of AAH, which is often followed by invasive adenocarcinoma.

**Figure 1:**
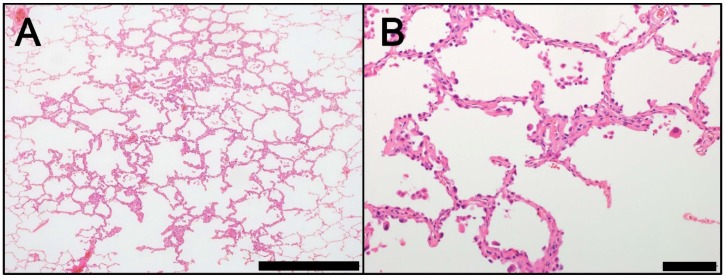
Morphology of AAH (hematoxylin and eosin staining). (**A**) Low magnification (Scale bar = 1000 µm). (**B**) High magnification (Scale bar = 100 µm). AAH, atypical adenomatous hyperplasia.

AAH is a small (usually smaller than 0.5 cm) lesion that typically occurs in the peripheral lung, especially near the pleura [1,3,13]. AAH is frequently undetectable macroscopically but can often be identified microscopically. Macroscopically identified AAH usually appears as a small, poorly-defined, tan-yellow, nodular lesion [1]. Microscopically, AAH exhibits localized proliferation of alveolar type II pneumocytes with mild to moderate cellular atypia, which line pre-existing alveolar walls (lepidic growth) (Figure 1) [1]. Atypical alveolar type II pneumocytes typically have a hobnail appearance. Intranuclear eosinophilic inclusions are occasionally observed. Between the atypical type II pneumocytes, substantial gaps are evident along the surface of the basement membrane. These gaps are useful characteristics to distinguish AAH from AIS (Figure 1). AAH lesions show an abrupt transition to adjacent normal pneumocytes. Although the alveolar wall may be slightly thickened by collagen, these interstitial changes do not extend beyond the borders of the lesion, as defined by the population of atypical pneumocytes [1].

AAH must be distinguished from AIS and reactive pneumocyte hyperplasia. AAH and AIS both present a morphological continuum of progression in terms of cellular and structural atypia. Therefore, the distinction between AAH with a greater number of cellular/structural atypia and AIS is occasionally challenging. In AIS, cellular/structural atypia are typically more pronounced than in AAH. It should be noted that AIS is generally larger (usually greater than 5 mm), with a greater population of densely packed atypical cells, with greater cell-cell contact, overlap, mild stratification, and abrupt transitions to adjacent normal pneumocytes [1]. Reactive pneumocyte hyperplasia with cellular atypia occurs secondary to parenchymal inflammation or fibrosis, where atypical pneumocytes are not the dominant feature. Typically, AAH does not occur in inflammatory or fibrotic lesions [1].

### 2.2. AIS (Adenocarcinoma In Situ)

**Figure 2:**
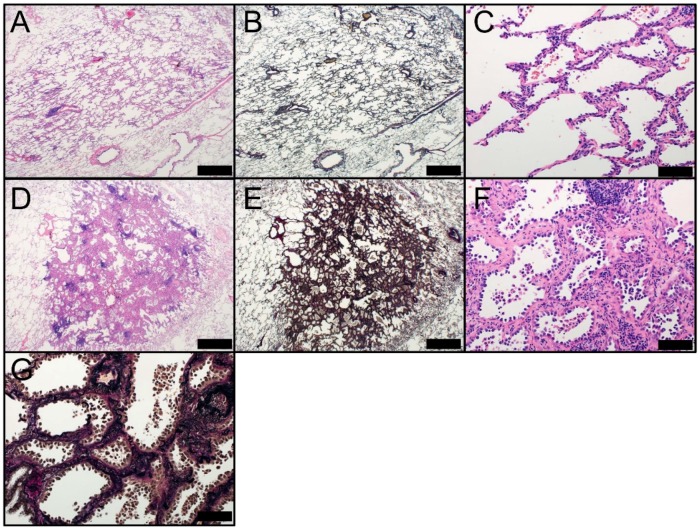
Morphology of AIS (Noguchi type A; **A**–**C**) and AIS, sclerosing type (Noguchi type B; **D**–**G**). AIS at low magnification (Noguchi type A) [(**A**) HE staining and (**B**) EVG staining] and (**C**) high magnification (HE staining). AIS, sclerosing type, at low magnification (Noguchi type B) [(**D**) HE staining and (**E**) EVG staining] and high magnification [(**F**) HE staining and (**G**) EVG staining]. **A**, **B**, **D**, and **E**: Scale bar = 1000 µm. **C**, **F**, and **G**: Scale bar = 100 µm. AIS, adenocarcinoma in situ; EVG, Elastic van Gieson; HE, hematoxylin and eosin.

AIS is a small (defined as less than or equal to 3 cm in diameter), localized adenocarcinoma that is typically identified in the lung periphery, close to the pleura [3,13]. In the Noguchi classification, the continuum from type A to type B, followed by type C, represents the stepwise progression of peripheral early adenocarcinoma of less than or equal to 2 cm in size [3,4]. AIS lesions, corresponding to Noguchi type A or type B adenocarcinoma, are localized and grow slowly. When AIS lesions are completely resected, disease-free survival is nearly 100% [14,15,16,17,18]. AIS tumors can be subdivided into nonmucinous and mucinous variants; however, the mucinous variant is extremely rare. AIS is usually identified coincidentally with the use of CT for other medical reasons. On CT, AIS appears as a small GGN with no solid component, similar to AAH. In cases of mucinous AIS, lesions very rarely show a part-solid or solid nodule [1,19]. Macroscopically, AIS typically appears as a poorly-demarcated nodule with a tan- or pale-colored cut surface. Microscopically, AIS appears as a pure lepidic growth of tumor cells along pre-existing alveolar structures and lacks lymphatic, vascular, stromal, or pleural invasion (Figure 2). The tumor should be completely sampled and microscopically examined to confirm the lack of an invasive component. Nonmucinous AIS typically comprises atypical type II pneumocytes (Figure 2A–C). Extremely rare cases of mucinous AIS consist of tall columnar cells with abundant cytoplasmic mucin and basal nuclei, resembling goblet cells. Nuclear atypia are virtually absent in mucinous AIS, whereas low-grade nuclear atypia are observed in nonmucinous AIS [1]. Immunohistochemically, nonmucinous AIS usually expresses TTF-1 (NKX2-1) protein, which is a transcription factor that regulates the tissue-specific expression of surfactant proteins [20] and a lineage-specific marker of terminal respiratory unit (TRU) [21,22,23]. In contrast, mucinous AIS rarely expresses TTF-1 protein; therefore, a majority of mucinous AIS tumors are considered non-TRU-type adenocarcinomas [23]. Both nonmucinous and mucinous AIS tumors show positive CK7 immunostaining. In contrast, mucinous AIS tumors are usually immunohistochemically positive for CK20, whereas nonmucinous AIS tumors rarely show such CK20-positive staining. The coexpression of CK7 and CK20 is a characteristic feature of mucinous lung adenocarcinoma, including mucinous AIS [24]. However, it should be noted that mucinous adenocarcinomas from other organs, such as ovaries, occasionally coexpress CK7 and CK20 [25]. Morphologically, AIS tumor cells typically resemble alveolar type II pneumocytes that grow with the continuous replacement of alveolar epithelium. AIS that lacks fibrotic foci and possesses a relatively thin stroma corresponds to Noguchi type A adenocarcinoma (Figure 2A–C). The sclerosing variant of AIS (corresponding to Noguchi type B adenocarcinoma) shows septal widening with alveolar collapse and an increase in elastic tissue (Figure 2D–G) [3,4]. Distinguishing between sclerosing AIS and invasive adenocarcinoma is occasionally challenging.

AIS must be distinguished from AAH (as described above) and MIA, which presents as a minimally invasive lesion. The delineation of invasive foci in MIA from sclerosing AIS with a marked increase of elastic tissue in thickened alveolar septa is potentially problematic. The use of an elastic tissue stain that demonstrates the destruction of alveolar structures allows the differentiation between true invasion and alveolar collapse [26,27]. However, it should be noted that the destruction of elastic fibers is not necessarily present during the early phase of invasion. Additionally, changes at the biopsy site from prior biopsies should not be misinterpreted as stromal reactions induced by tumor invasion [28]. Noninvasive adenocarcinomas greater than 3 cm in diameter are very rare; thus, there is insufficient evidence for a prognosis of 100% disease-free survival when they are removed completely. In the 2015 WHO classification, these tumors are classified as “lepidic predominant adenocarcinoma, suspect AIS” [1].

### 2.3. MIA (Minimally Invasive Adenocarcinoma)

**Figure 3:**
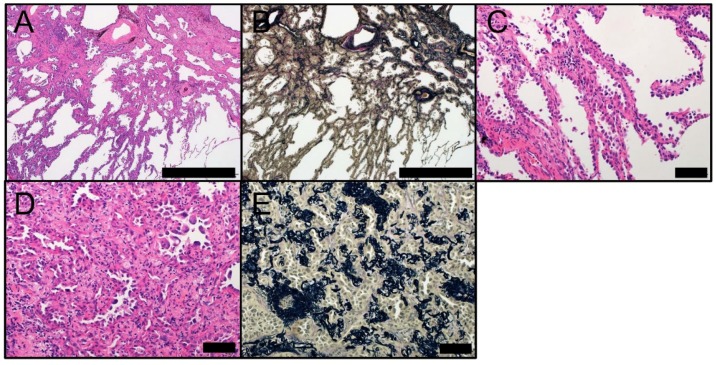
Morphology of MIA. MIA at low magnification ((**A**) HE staining and (**B**) EVG staining) (Scale bar = 1000 µm). Lepidic component of MIA at high magnification ((**C**) HE staining) and the invasive component ((**D**) HE staining and (**E**) EVG staining) (Scale bar = 100 µm). EVG, Elastic van Gieson; HE, hematoxylin and eosin; MIA, minimally invasive adenocarcinoma.

MIA has been introduced as a new tumor entity between AIS and lepidic adenocarcinoma in the 2015 WHO classification [1]. The introduction of MIA is logical from a clinicopathological point of view [27]. MIA is a small (less than or equal to 3 cm in diameter) adenocarcinoma with a predominantly lepidic pattern and an invasive structure smaller than or equal to 5 mm (Figure 3). MIA is usually nonmucinous but rarely can be mucinous [1]. As with AIS, MIA is usually detected coincidentally on CT performed for other medical reasons. On CT, MIA frequently appears as a GGN in the lung periphery with a solid component smaller than 5 mm. The invasive lesion requires differentiation from alveolar collapse, fibrosis, and mucous that can also cause a solid appearance on CT. Whereas the differential diagnosis between MIA and mucinous AIS is problematic on CT, multiple nodules and spread of the nodule to adjacent lung parenchyma with an indistinct border support the diagnosis of mucinous AIS [29]. Clinically, MIA is not expected to recur if removed completely [14,17,18,30,31,32]. As previous studies have not demonstrated any significant differences between AIS and MIA in terms of clinical outcome, the benefit of their differentiation is questionable [33,34]. Based on previous research [1,2], neither AIS nor MIA tend to spread to regional lymph nodes or metastasize. Therefore, patients with AIS or MIA are candidates for sublobar resection [35,36,37,38].

Macroscopically, MIA typically appears as a centrally elastic-hard, fibroblastic lesion surrounded by an elastic-soft, pneumatic component. MIA lesions frequently exhibit central anthracotic pigmentation and pleural puckering. Tumor size on gross examination may be smaller than that on radiological examination. Collapse of the lung tissue, especially when the lung is not adequately filled with formalin, and formalin fixation itself may contribute to the reduced gross size. Furthermore, ill-demarcated tumor, because of the sparse lepidic growth in the periphery, may lead to a smaller macroscopic size compared to radiology. Microscopically, MIA appears as an adenocarcinoma (less than or equal to 3 cm) with a predominant lepidic pattern and invasive structure less than or equal to 5 mm (Figure 3) [1,39]. The size of the invasive area must be correctly measured in the largest dimension, based on classification criteria [1,39]. Scattered invasive foci make it difficult to measure the greatest diameter of the structure. A recent study suggested that the size of the invasive structure can be estimated as the sum of the percentages of the invasive components multiplied by the greatest diameter of the tumor [1,14]. According to the classification criteria, a small (less than or equal to 3 cm) adenocarcinoma with an estimated invasive diameter less than or equal to 5 mm can be diagnosed as MIA [1,14]. Microscopically, MIA appears nonmucinous but very rarely can be mucinous or of a mixed phenotype, similar to AIS [1,30,32,40]. If the tumor size is less than or equal to 2 cm, MIA is classified as a Noguchi type C tumor. Noguchi type C adenocarcinoma represents localized adenocarcinoma with lepidic growth and foci of active fibroblast proliferation [3,4]. The invasive component of MIA can display various histological patterns, such as acinar, papillary, solid, or micropapillary. The infiltration of tumor cells into the stroma with active myofibroblast proliferation is also considered invasive. MIA is defined as lacking lymphatic, vascular, and pleural invasive components; tumor necrosis; or spreads through air spaces, all of which are associated with a poor outcome. Immunohistochemically, nonmucinous MIA tumors are TRU-type adenocarcinomas that stain positive for pneumocyte markers TTF-1 and Napsin A, which is an aspartic proteinase involved in the maturation of surfactant protein B in type II pneumocytes [21,41]. Mucinous MIA tumors are frequently negative for pneumocyte-associated proteins and positive for CK20 and HNF4A [42], which is a transcription factor expressed in the liver, kidney, and intestine that plays a crucial role in morphological differentiation [43]. HNF4A is frequently detected in invasive mucinous adenocarcinomas [42].

MIA must be properly distinguished from AIS (as described above) and invasive lepidic adenocarcinoma. When scattered invasive foci are observed in a tumor, measuring or estimating the size of the invasive component correctly is challenging; however, this step is necessary to make a proper differential diagnosis. It should be noted that invasive lepidic adenocarcinoma resembles MIA in terms of morphological features but has a significantly worse clinical outcome than MIA. Acinar and papillary patterns are the most commonly observed invasive components in MIA [14]; therefore, the architectural similarity of these patterns with lepidic patterns makes it more difficult to differentiate between a noninvasive lepidic pattern and an invasive pattern [44]. An intraoperative diagnosis using frozen sections may be used to stratify lung tumors into AIS, MIA, and overt invasive adenocarcinoma, which may help determine treatment strategies (lobectomy or sublobar resection) [45]. The accuracy of an intraoperative diagnosis is dependent on multiple factors, such as tumor size, interstitial inflammation or fibrosis, number of pathologists engaging in the diagnosis, and number of examined samples [45,46,47,48]. As most studies of adenocarcinomas with minor invasive components involved the examination of tumors smaller than 3 cm, there is insufficient evidence for a prognosis of 100% disease-free survival when such tumors are removed completely. Therefore, these tumors are classified as “lepidic predominant adenocarcinoma, suspect MIA” in the 2015 WHO classification [1].

## 3. Molecular Pathogenesis through a Stepwise Continuum

The multistep progression of lung adenocarcinoma is supported by the molecular and clinicopathological features of the tumor. The progression from AAH to AIS, followed by invasive adenocarcinoma, is based on the molecular evidence described below.

### 3.1. AAH

AAH is the presumed lesion from which lung adenocarcinoma arises. The premalignant nature of AAH is supported by a variety of molecular findings that show similarities between AAH and adenocarcinoma. AAH demonstrates clonality [49,50], loss of heterozygosity of chromosomes, including 9q and 16p [51], mutations of *EGFR*, *BRAF*, *KRAS*, *FGFR3*, and *ERBB2* [52], high expression levels of *CCND1* (*cyclin D1*), and low expression levels and epigenetic downregulation of *CDKN2A* (*p16*) [53,54]. AAH harbors some molecular alterations observed in lung adenocarcinoma, which supports the progression from AAH to adenocarcinoma. These studies have identified several aspects of pathogenesis of AAH; however, the complex nature of its molecular pathology is still poorly understood and must be further elucidated.

### 3.2. AIS

In the multistep continuum, AIS is the intermediate step between AAH and MIA [1]; however, little evidence exists for the molecular events that allow the progression from AAH to AIS. Aberrant DNA methylation gradually increases along the continuum [55]. Specific CpG islands are significantly hypermethylated in AIS compared to those in AAH [55]. Telomere shortening and DNA damage responses (DDRs) are considered an early event in lung carcinogenesis. Expression levels of DDR proteins and mRNAs for *TERF1* and *TERF2* that encode telomere-specific DNA-binding proteins are associated with the progression from AAH to AIS [56,57]. Driver genetic mutations, including *EGFR*, *KRAS*, and *BRAF*, are thought to be associated with the initiation and progression of adenocarcinoma as described in this review.

### 3.3. MIA

MIA is an early invasive adenocarcinoma; therefore, its genetic alterations are associated with molecular events that allow invasion [1]. The invasiveness of lung adenocarcinoma via the multistep continuum has been associated with multiple molecular events. For adenocarcinomas containing *EGFR* mutations, *EGFR* amplification is involved in the transition from AIS to MIA [58,59,60]. *TTF-1* amplification typically occurs coincident with *EGFR* amplification [59,61,62]. The frequency of *TP53* mutations increases during the progression to invasive tumors [32,63,64]. The number of regions of allelic imbalance is higher in invasive tumors [63]. Repression of *TGFBR2* acts as a determinant of invasiveness [65]. *MiR-9-5p* promotes invasion by downregulating *TGFBR2* expression [66]. Amplification and overexpression of *PDCD6* and *TERT* on chromosome 5p promote invasion [67]. Laminin-5, an invasion-associated molecule [68,69], is activated during the progression from AIS to MIA.

## 4. Driver Mutations in AAH, AIS, and Invasive Adenocarcinoma

The EGFR-RAS-RAF-MEK (MAP2K)-ERK (MAPK1) signaling pathway is a key regulator of cell growth and transformation [70]. When activated, this signaling pathway leads to uncontrolled cell proliferation, increased cell survival, and tumor initiation and progression. EGFR is a receptor tyrosine kinase belonging to the ERBB family, whereas KRAS is an important effector of activated EGFR. The *KRAS* oncogene belongs to the *RAS* family of genes that encode guanosine-5′-triphosphate-binding proteins. *BRAF*, a member of the *RAF* gene family, encodes a serine-threonine protein kinase that is a downstream effector of activated RAS, activating MEK (MAP2K) and leading to tumor initiation and progression via the activation of ERK (MAPK1).

### 4.1. EGFR and KRAS

Sakamoto et al. examined *EGFR* and *KRAS* mutations in 119 synchronous pulmonary lesions, including 40 cases of AAH, 26 cases of AIS, 14 cases of MIA, and 34 cases of overt invasive adenocarcinoma [71,72]. The mutually exclusive nature of *EGFR* and *KRAS* mutations was maintained, even in preinvasive lesions (AAH and AIS), similar to invasive adenocarcinomas [71,72,73]. Of note, these authors demonstrated that the rate of *KRAS* mutations decreased along the stepwise continuum: 33% in AAH, 12% in AIS, 8% in MIA, and 0% in well-differentiated invasive adenocarcinoma. In contrast, the frequency of *EGFR* mutations was distributed in a similar proportion along the multistep continuum. Interestingly, moderately and poorly differentiated adenocarcinomas harbored *KRAS* mutations (18% and 17% respectively) more frequently than well-differentiated adenocarcinomas, suggesting potential de novo carcinogenesis as a result of *KRAS* mutations and additional genomic events. In terms of smoking status, *KRAS*-mutated AAH and adenocarcinoma were more frequently observed in smokers [71,72]. As for *EGFR* mutations, while there is a significant association between *EGFR* mutations and nonsmoking in invasive adenocarcinoma, one-half of AAH lesions developed in smokers [71]. The relatively high frequency of *KRAS* mutations in AAH lesions in smokers is consistent with the results of recent high-throughput studies [52,74,75]. We also examined the association between smoking status and molecular features in 110 AIS lesions [76]. We demonstrated that mutation rates of *EGFR* and *KRAS* did not differ by smoking status in AIS lesions. In contrast, mutations in *EGFR* and *KRAS* were significantly associated with smoking status in invasive adenocarcinoma: *EGFR* mutations in nonsmokers and *KRAS* mutations in smokers [76], consistent with findings from previous studies [77,78]. These results suggest that AIS may be a distinct biological entity of lung adenocarcinoma, and that smoking may act not only as a cause of AIS but also as a promoter of invasion [76].

### 4.2. BRAF

Emerging evidence demonstrates that AAH and AIS lesions frequently harbor *BRAF* mutations [52,74,75], despite the low frequency of *BRAF* mutations in invasive adenocarcinoma. According to a study by Sivakumar et al. [75], *BRAF* was the most commonly mutated gene in AAH (23%), followed by *KRAS* (18%). Whereas *KRAS* mutations in AAH were associated with smokers, *BRAF* mutations were not associated with a smoking history [75], which is consistent with results from another study [52]. Of interest, no *BRAF* mutations were found in lung adenocarcinomas that coexisted with *BRAF*-mutated AAH lesions, whereas four of five cases of *BRAF*-mutated AAH tumors coexisted with *EGFR*-mutated adenocarcinomas [75]. Among studied AAH lesions, *BRAF* mutations are mutually exclusive of *KRAS* mutations. These results are also consistent with those of similar studies [52,74].

### 4.3. EGFR vs. KRAS and BRAF

**Figure 4:**
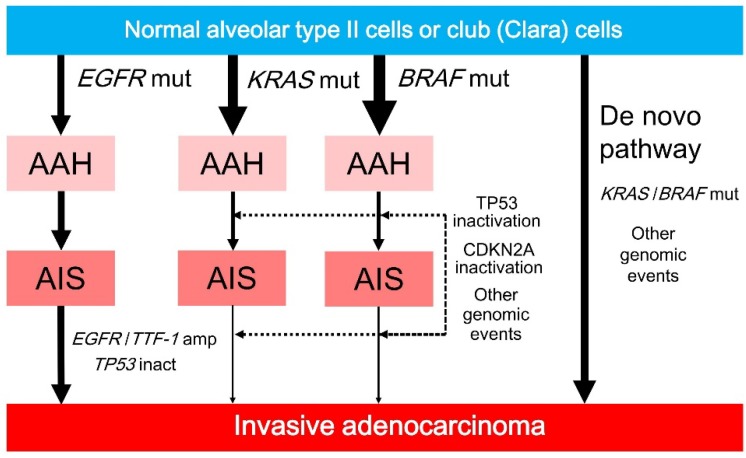
AAH develops from normal alveolar type II cells or club (Clara) cells after mutations in *EGFR*, *KRAS*, and *BRAF*. *EGFR*-mutated AAH progresses to AIS, followed by invasive adenocarcinoma after *EGFR*/*TTF-1* amplification. *KRAS*- or *BRAF*-mutated AAH rarely progresses to AIS or invasive adenocarcinoma, but can do so after the inactivation of TP53/CDKN2A (p16) or other genomic events (dotted arrows). *KRAS*- or *BRAF*-mutated invasive adenocarcinoma may arise from normal lung epithelium via a de novo pathway that involves *KRAS* or *BRAF* mutations and other genomic events. Solid arrows indicate progression. AAH, atypical adenomatous hyperplasia; AIS, adenocarcinoma in situ; amp, amplification; inact, inactivation; mut, mutation.

Of interest, the frequencies of *KRAS* and *BRAF* mutations are higher in preinvasive lesions than in invasive lesions, whereas the frequency of *EGFR* mutations in preinvasive lesions is similar to that in invasive lesions (Figure 4). These results suggest that *KRAS* or *BRAF* mutations induce cell proliferation and cellular atypia; however, preinvasive lesions containing *KRAS* or *BRAF* mutations rarely progress to invasive lesions unless they undergo additional genomic alternations (Figure 4). Preinvasive lesions containing *KRAS* mutations may become invasive after additional genomic mutations caused by smoking, because of a well-known association between *KRAS* mutations and smoking in invasive adenocarcinoma. Preinvasive lesions containing *BRAF* mutations may also become invasive after additional genomic alterations. However, more plausible explanations include de novo developments from normal lung epithelium to *KRAS*- or *BRAF*-mutated invasive adenocarcinomas as a result of *KRAS* or *BRAF* mutations and additional genomic changes, including genomic instability caused by defective DNA repair or smoking (Figure 4) [79,80].

## 5. Driver Mutations in Lung Tumorigenesis

The comparative associations between the frequencies of *EGFR*, *KRAS*, and *BRAF* mutations in preinvasive versus invasive lesions may be explained by experiments using mice that consistently express these mutants [72,81,82,83,84].

### 5.1. EGFR

*EGFR*-mutated adenocarcinoma is characterized by East-Asian ethnicity, female gender, non/light-smoking history, and hobnail cell morphology [85,86]. As *EGFR*-mutated adenocarcinomas frequently develop via the presumed multistep continuum [52,71,74,75,76], incidence rates of AAH, AIS, and MIA are high in East-Asian populations. Mutations and amplification of *EGFR* are closely associated with each other, and *EGFR* amplification occurs only in *EGFR*-mutated adenocarcinoma [87,88]. In the stepwise development of *EGFR*-mutated adenocarcinoma, *EGFR* amplification plays a crucial role in the progression to invasive adenocarcinoma [58,60].

Ji et al. generated bitransgenic mice in which the expression of two common *EGFR* mutations (i.e., exon 19 deletion and L858R point mutation in exon 21) could be induced in alveolar type II pneumocytes [81,89,90]. After the continuous expression of *EGFR* mutants, the bitransgenic mice developed invasive lung adenocarcinoma via the multistep continuum: the progression from AAH to AIS, followed by invasive adenocarcinoma with lepidic features [81]. These authors demonstrated that the continual expression of the *EGFR* mutant is not only essential for tumor development but also for tumor stability, and that *EGFR*-targeted therapy for *EGFR*-mutated lung adenocarcinoma is dramatically effective, suggesting that *EGFR* mutants are directly involved in tumor maintenance.

### 5.2. KRAS

Studies using human preinvasive and invasive lesions demonstrated that *KRAS* mutations are observed more frequently in preinvasive lesions than in invasive lesions [52,71,74,75,76]. These discordant frequencies may be explained by experiments using mice that continuously expressed a *KRAS* mutant. Johnson et al. demonstrated that active somatic mutations of the *KRAS* oncogene cause early onset lung tumors in mice [82]. The *KRAS* mutant caused a benign, noninvasive lung alveolar tumor, closely resembling AAH. Only the selected cell lineage (alveolar type II pneumocytes, not club (Clara) cells) was affected by the expression of the *KRAS* mutant, and a benign alveolar tumor developed to invasive adenocarcinoma via TP53 inactivation [82]. Collado et al. showed that benign-looking bronchioloalveolar tumors with *KRAS* mutations in mice harbored biological features that were distinct from those observed in invasive adenocarcinoma [84]. Benign-looking tumors with *KRAS* mutations underwent oncogene-induced senescence, as shown by the expression of senescence-associated β-galactosidase [91] and the presence of senescence-associated heterochromatin foci [84,92]. These authors concluded that a substantial number of cells in *KRAS*-mutated benign-looking tumors undergo oncogene-induced senescence, but that *KRAS*-mutated invasive adenocarcinomas do not undergo senescence due to the inactivation of oncogene-induced senescence effectors such as TP53 and CDKN2A [84]. As for *KRAS* mutations in colorectal tumors, Bennecke et al. demonstrated that murine intestinal epithelial cells with *KRAS* mutations developed serrated hyperplasia, which is characterized by CDKN2A overexpression and induction of senescence [93]. However, a *CDKN2A* deletion in mutant *KRAS*-expressing mice prevented the progression to senescence and led to invasive and metastasizing adenocarcinomas with morphological and molecular features similar to *KRAS*-mutated adenocarcinomas. Cellular transformation induced by mutant *KRAS* is not sufficient to drive carcinogenesis; *KRAS*-mutated cells require other genomic events, including genomic instability in the context of the inactivation of the TP53 pathway [79,80]. Other mechanisms of *KRAS*-induced carcinogenesis include increases in levels of reactive oxygen species (ROS), and the association of ROS generation with malignant cellular transformation [94]. The mechanism underlying *KRAS*-driven carcinogenesis remains unclear because of the complexity of its downstream effectors [80,95]. Collectively, although precise mechanisms are still unclear, *KRAS* mutations play divergent roles in both cellular senescence and carcinogenesis.

In 2017, Vaz et al. showed that exposure to chronic cigarette smoke induces progressive epigenetic alterations of bronchial epithelial cells that sensitize these cells to transformation with a single *KRAS* mutation, eventually driving the development of lung cancer [96]. Their results provide a paradigm in which epigenetic alterations may precede (and sensitize cells to) genetic events that drive lung carcinogenesis. Methylated gene abnormalities greatly overlap those commonly seen in smokers with lung cancer. Chronic exposure to cigarette smoke causes the hypermethylation of genes that play significant roles in the regulation of WNT signaling (*SFRP2*, *SFRP5*, and *WIF1*), the apoptotic function of TP53 (*MSX1*), and RAS/MAPK signaling (*BMP3*, *WIF1*, and *GATA4*) [96,97]. These epigenetically altered pathways, including the RAS/MAPK [98] and WNT [99,100,101,102] pathways, can potentially drive cigarette smoke-induced lung cancer [103,104]. Taken together, smoking induces epigenetic alterations that make cells vulnerable to key genetic events, such as *KRAS* mutations, driving carcinogenesis. Thereafter, altered pathways or other genomic events may promote tumor progression.

### 5.3. BRAF

The *BRAF^V600E^* mutation causes oncogene-induced senescence [105], similar to *KRAS* mutation-induced senescence [84]. The relevance of *BRAF* mutations in AAH and invasive adenocarcinoma corresponds to that of melanocytic nevus and malignant melanoma, both of which carry the oncogenic *BRAF^V600E^* mutation [105]. Melanocytic nevus is a commonly observed benign tumor of melanocyte origin [106]. Although nevi frequently harbor the oncogenic *BRAF^V600E^* mutation [107], the initial step of nevus growth is typically followed by growth arrest from oncogene-induced senescence that blocks *BRAF^V600E^*-mediated oncogenic signaling. Growth-inhibitory responses induced by the *BRAF^V600E^* mutant show classical hallmarks of senescence (i.e., induction of both CDKN2A and senescence-associated acidic β-galactosidase activity). In contrast, nevi with the *BRAF^V600E^* mutation develop into malignant melanomas via the inactivation of the TP53 pathway or CDKN2A [108,109].

In 2017, Nieto et al. identified the role of a kinase-inactive *BRAF* mutation in the tumorigenesis of lung adenocarcinoma [110]. The oncogenic mechanism of activating *BRAF* mutations, such as *BRAF^V600E^*, is known to promote MEK-ERK activation; however, the biological role of kinase-inactive *BRAF* mutations, which are more common in lung adenocarcinoma than the activating *BRAF^V600E^* mutation [111,112], is not known. On the other hand, inactivating *BRAF* mutations have been identified in a subset of *KRAS*-activated lung cancers. These authors demonstrated that the expression of an endogenous kinase-inactive *BRAF* mutant triggered the development of lung adenocarcinoma in mice, indicating that *BRAF*-inactivating mutations initiate lung oncogenesis. Furthermore, the coexpression of activating *KRAS* mutations and inactivating *BRAF* mutations in mouse lung cells markedly enhanced tumor initiation, a phenomenon mediated by CRAF kinase activity [113,114], and effectively accelerated tumor progression to advanced lung adenocarcinomas. Their results also suggest that the signal intensity of the MAPK pathway is a critical determinant not only of tumor development, but also of tumorigenesis [110].

## 6. Conclusions and Future Directions

This review summarizes the state of knowledge of tumorigenesis and progression of early lung adenocarcinoma, with a special focus on its clinicopathological characteristics and their associations with driver mutations (*EGFR*, *KRAS*, and *BRAF*). Most cases of lung adenocarcinoma are found at advanced stages; therefore, improved detection at curable stages is needed to decrease the number of disease-associated deaths. The most prevalent subset of adenocarcinoma, including *EGFR*-mutated adenocarcinoma, appears to develop from AAH to AIS, to MIA, to overt invasive adenocarcinoma with a lepidic pattern, which is typically observed in TRU-type adenocarcinoma. In contrast, oncogene-induced senescence is likely to reduce the frequency of the stepwise progression in *KRAS*- or *BRAF*-mutated adenocarcinoma. Oncogene-induced senescence, epigenetic alterations, or kinase-inactive mutation may be associated with the tumor evolution of *KRAS*- or *BRAF*-mutated adenocarcinoma. While the mechanism of tumorigenesis in lung adenocarcinoma is unclear, a better understanding of tumorigenesis and progression in such cases may allow the development of effective preventive, screening, and therapeutic strategies.

## Abbreviations

AAH	atypical adenomatous hyperplasia
AIS	adenocarcinoma in situ
CT	computed tomography
DDR	DNA damage response
EVG	Elastic van Gieson
GGN	ground-glass nodule
HE	hematoxylin and eosin
MIA	minimally invasive adenocarcinoma
ROS	reactive oxygen species
TRU	terminal respiratory unit
WHO	World Health Organization
